# Seizure-Induced Acute Glial Activation in the *in vitro* Isolated Guinea Pig Brain

**DOI:** 10.3389/fneur.2021.607603

**Published:** 2021-01-26

**Authors:** Diogo Vila Verde, Marco de Curtis, Laura Librizzi

**Affiliations:** Epilepsy Unit, Fondazione IRCCS Istituto Neurologico Carlo Besta, Milano, Italy

**Keywords:** blood brain barrier, inflammation, albumin, microglia, astrocytes

## Abstract

**Introduction:** It has been proposed that seizures induce IL-1β biosynthesis in astrocytes and increase blood brain barrier (BBB) permeability, even without the presence of blood borne inflammatory molecules and leukocytes. In the present study we investigate if seizures induce morphological changes typically observed in activated glial cells. Moreover, we will test if serum albumin extravasation into the brain parenchyma exacerbates neuronal hyperexcitability by inducing astrocytic and microglial activation.

**Methods:** Epileptiform seizure-like events (SLEs) were induced in limbic regions by arterial perfusion of bicuculline methiodide (BMI; 50 μM) in the *in vitro* isolated guinea pig brain preparation. Field potentials were recorded in both the hippocampal CA1 region and the medial entorhinal cortex. BBB permeability changes were assessed by analyzing extravasation of arterially perfused fluorescein isothiocyanate (FITC)–albumin. Morphological changes in astrocytes and microglia were evaluated with tridimensional reconstruction and Sholl analysis in the ventral CA1 area of the hippocampus following application of BMI with or without co-perfusion of human serum albumin.

**Results:** BMI-induced SLE promoted morphological changes of both astrocytes and microglia cells into an activated phenotype, confirmed by the quantification of the number and length of their processes. Human-recombinant albumin extravasation, due to SLE-induced BBB impairment, worsened both SLE duration and the activated glia phenotype.

**Discussion:** Our study provides the first direct evidence that SLE activity *per se* is able to promote the activation of astro- and microglial cells, as observed by their changes in phenotype, in brain regions involved in seizure generation; we also hypothesize that gliosis, significantly intensified by h-recombinant albumin extravasation from the bloodstream to the brain parenchyma due to SLE-induced BBB disruption, is responsible for seizure activity reinforcement.

## Introduction

Blood brain barrier (BBB) dysfunction has been associated with disturbances of neural function in the central nervous system (CNS). A compromised BBB is often found in epileptic brain tissue obtained from epilepsy surgery (1, 2) and in patients with post-traumatic epilepsy (3) as well as in pharmacological models of status epilepticus (4). Experimental BBB leakage during intense seizures and the associated extravasation of serum albumin have been recognized as important contributors to glial dysfunction and epileptogenesis (5–7). Serum albumin binds the transforming growth factor ß (TGF-ß) receptor II in astrocytes and activates a transcriptional response resulting in a cascade of events culminating in the generation of epileptiform discharges (8, 9). Accumulating data from human and animal studies support the notion that glial cells make an important contribution to the pathogenesis of neurological diseases (10–12). Astrocytes are indispensable for proper brain development, playing fundamental roles in promoting formation and function of synapses, maintaining ion, neurotransmitter, water and ATP homeostasis and modulating neuronal signaling (13–16). Astrocytes can become reactive and develop a gliosis-like state where inflammation processes are triggered and up-regulated in a positively-feedback loop after brain injury and disease (17, 18), having a key role in in the generation and spread of seizure activity (11, 19–21).

Through dedicated molecular cascades, astrocytes (i) protect neurons against glutamate excitotoxicity by removing and recycling this neurotransmitter released during neuronal activity from the extracellular space; (ii) remove extracellular activity-dependent potassium accumulation; (iii) reduce the subsequent neuronal depolarization and hyperactivity (22, 23). Astrocytes also represent an important source of pro-inflammatory mediators and have been shown to initiate and regulate many immune-mediated mechanisms in the CNS (24–26). Changes of astrocytic receptors, transporters, ion channels and intracellular proteins are present in almost all forms of epilepsy (27). Accordingly, modified astroglial functioning is a key element leading to a reduction in: (i) expression of potassium inward-rectifying channels (Kir4.1) and water channels (aquaporin 4, AQP4) resulting in impaired potassium [K^+^]_o_ buffering, [K^+^]_o_ accumulation and consequent neuronal depolarization and seizures (28, 29); (ii) gap junction expression, with consequent alteration of spatial buffering of small molecules (e.g., K^+^) (30, 31); (iii) glutamate uptake, favoring brain excitability increase (11).

Microglial cells are brain-resident macrophage-like cells that contribute to innate immune system mechanisms and respond early to CNS injuries (32). Accordingly, their reaction to damage can be either detrimental or protective (33). In a resting state, microglial cells feature a small cell body with vastly ramified processes (surveilling microglia). After a pathological challenge, microglial cells acquire amoeboid-like shape somata with almost no processes and achieve phagocytic properties (32). Recently, resident microglial cells have been implicated in driving astrocytes reactivity (34, 35) contributing to neuronal hyperexcitability and neurodegeneration (36, 37) and to the process of epileptogenesis in human and animal models of epilepsy (38, 39). Serum albumin-activated microglia releases pro-inflammatory cytokines [TNF-α; (40, 41)] and interacts with the damage-associated molecular patterns [DAMPs; (42)], contributing to astrocytes activation, brain inflammation and seizure recurrence (24, 40, 43). Seizures by themselves can induce brain inflammation and gliosis independent from blood-borne molecules, mediated by the synthesis and release of IL-1β that promotes BBB disruption (44).

In this study, we aim to investigate more in detail the effects of seizure activity on glial response, focusing on the morphological changes characterizing reactive glial cells. We also investigate if brain parenchyma exposure to serum albumin worsens glial cells reactivity and, as consequence, favors brain excitability and seizure recurrence. To verify these hypotheses, we induced pharmacological seizures in the *in vitro* isolated guinea pig brain (44, 45), a preparation that retains the physiological interactions between neurons, glia and vascular compartments (BBB included) in a condition close to *in vivo* (46, 47). In this isolated preparation, seizure-induced inflammatory responses can be analyzed in the absence of peripheral immune cells or blood-derived molecules.

## Materials and Methods

Procedures involving animals and their care were conducted in accordance with the ethically approved institutional guidelines that are in compliance with national and international laws and policies (European Economic Community Council Directive 86/609, Official Journal L 358, 1, December 12, 1987; Guide for the Care and Use of Laboratory Animals, U.S. National Research Council, 1996). All efforts were made to minimize the number of animals used and their suffering. Brains were isolated from young adult Hartley guinea pigs (150–200 g; Charles River Laboratories, Comerio, Italy) according to the standard technique described in detail elsewhere (46, 48). After barbiturate anesthesia the brain was carefully isolated and transferred to the incubation chamber. The basilar artery was cannulated with a polyethylene cannula to ensure arterial perfusion with a saline solution (composition: NaCl, 126 mM, KCl, 3 mM, KH_2_PO_4_, 1.2 mM, MgSO_4_, 1.3 mM, CaCl_2_, 2.4 mM, NaHCO_3_, 26 mM, glucose, 15 mM, 3% dextran M.W. 70,000) oxygenated with a 95% O_2_-5% CO_2_ gas mixture (pH 7.3). This solution was arterially perfused at a rate of 6.5 ml/min via a peristaltic pump (Gilson Minipulse, Villiers Le Bel, France). Brain isolation was performed at low temperature (15°C) and experiments were carried out at 32°C, to maintain the isolated brain under hypothermic anesthesia. Human recombinant albumin (h-ALB; Sigma-Aldrich, Italy; 1 gr/250 ml) and bicuculline methiodide (Sigma-Aldrich, Italy) were applied by arterial perfusion (49, 50).

### Induction of Epileptiform Activity

**Figure 1:**
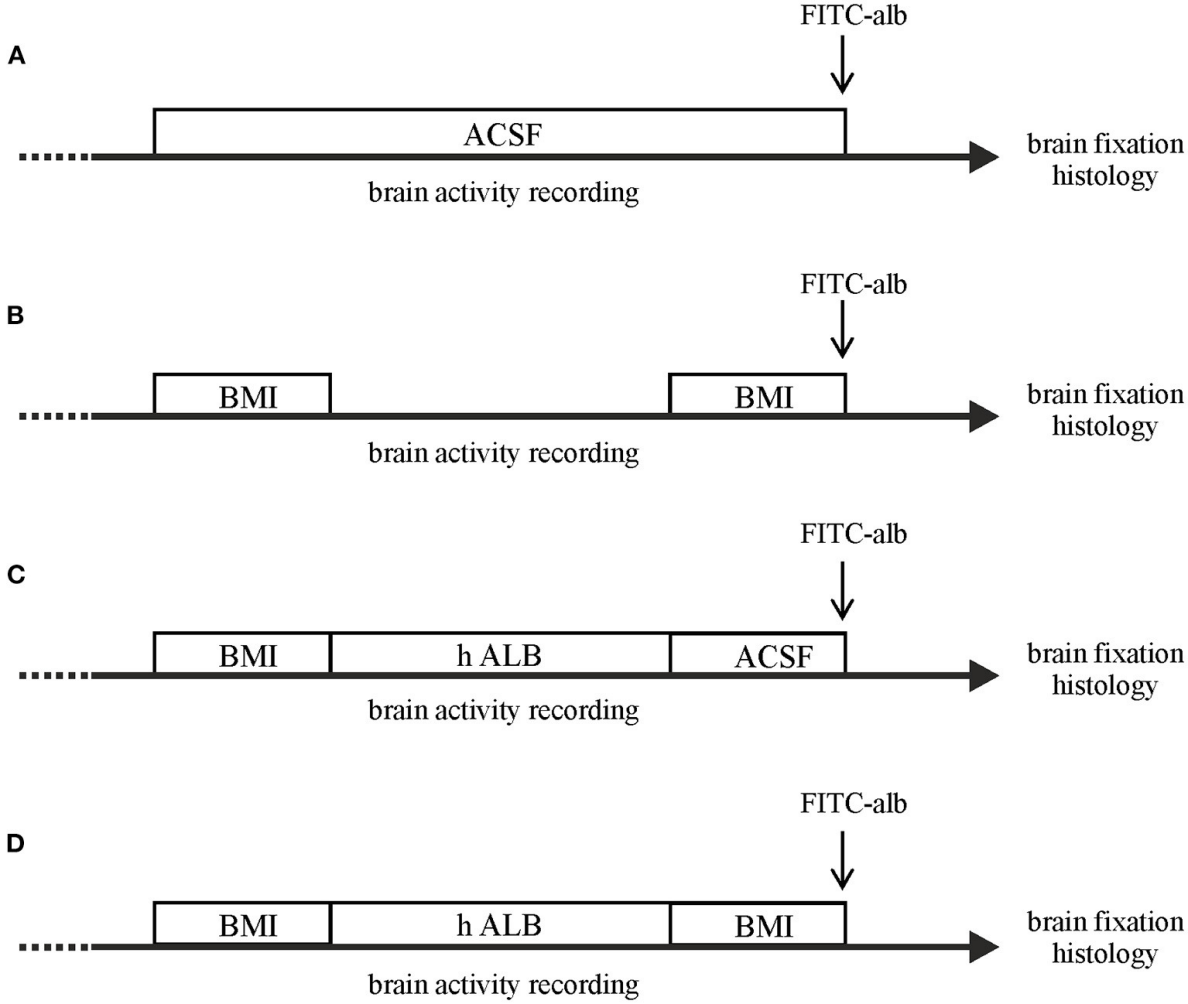
Schematic drawing of the different experimental protocols utilized in the study. The *in vitro* brains were arterially perfused with: **(A)** control saline solution; **(B)** a double 3-min bolus of BMI; **(C)** 30-min perfusion with h-recombinant albumin (h-ALB) after the birst bolus of BMI; **(D)** 30-min perfusion with h-recombinant albumin (h-ALB) interposed between two boli of BMI. At the end of each experiment the brain was perfused with FITC-albumin for 4 min to evaluate BBB leakage. Each experimental protocol lasted 4 h.

In a first set of experiments, epileptiform seizure-like events (SLEs) were induced by arterial perfusion of the GABA_A_ antagonist BMI (50 μM; *n* = 4) and a second BMI perfusion was applied 90 min after the first one (Figure 1, protocol B). In a second set of the experiments, h-ALB (4 g/L, 329 mOsm; *n* = *3*) added to control solution was arterially perfused for 30 min after the first bolus of BMI (Figure 1, protocol C). In a third set of the experiments, h-ALB (*n* = *4*) added to control solution was arterially perfused for 30 min, between the two BMI applications (Figure 1, protocol D), just after the recovery of the first SLE. Brains were maintained *in vitro* for 4 h. In control experiments, brains were perfused only with control saline solution (Figure 1, protocol A; *n* = *4*). Two control brains were perfused with FITC-albumin at the end of the experiment.

### Electrophysiology

To test brain viability during the experiments, simultaneous extracellular recordings were performed in the piriform cortex (PC), medial entorhinal cortex (mEC), and the CA1 hippocampal region with glass micropipettes filled with a 0.9% NaCl solution (2–5 MOhm input resistance) during stimulation of the lateral olfactory tract with bipolar twisted-wire silver electrodes (51, 52).

### Evaluation of BBB Permeability

The morphological and functional integrity of the BBB in the *in vitro* isolated guinea pig brain preparation has been previously demonstrated (47). We assessed the presence of BBB breakdown in isolated brains by perfusing fluorescein-isothiocyanate (FITC)–albumin (50 mg/10 ml, Sigma-Aldrich, Italy; *n* = *9*) for 4 min immediately before the brains were fixed for histologic analysis. Control brains (*n* = *4*) were maintained *in vitro* for a comparable time as experimental brains. The brief FITC-alb perfusion at the end of the experiment was utilized as a fluorescent marker of protein extravasation; the prolonged non-fluorescent h-ALB perfusion was used to enhance tissue excitability.

### Immunohistochemistry

At the end of the electrophysiological experiments, brains were removed from the recording chamber and were fixed by immersion into 4% paraformaldehyde in phosphate-buffered saline (PBS; 0.1M, pH 7.4) for at least 24 h; 50 μm thick coronal sections were cut by vibratome (VT 1000S Leica, Heidelberg, Germany) throughout the extension of the hippocampus (plates A5.4–A7.4 of guinea pig brain atlas by Luparello). Sections collected on gelatin-coated slides were mounted in Fluorosave (Calbiochem, San Diego, CA, U.S.A.), and were cover-slipped. Two sections corresponding to plates A5.40 and A5.76 were collected for each brain to assess the intraluminal vs. extravascular FITC–albumin fluorescein signal. Slide-mounted sections were examined with a laser scanning confocal microscope using excitation of 488 nm (Laser Ar). Quantification of parenchymal FITC–albumin was performed in the hippocampal formation. In each brain, three high -power non-overlapping fields per section were acquired bilaterally at 10x magnification. Laser intensity was set at 30–35% power. Gain and photomultiplier were kept constant during acquisition of all images. As index of BBB damage, the area (number of pixels) occupied by the extravascular parenchymal FITC signal was quantified. Data obtained were used for statistical analysis. Values for experimental groups were expressed as percentage of the mean leakage area in the control group (defined as 100%). A standardized protocol was used for histochemical staining: in short, after endogenous peroxidase inactivation (3% H_2_O_2_ in PBS) and non-specific antigen binding sites blocking (1% BSA/0.2% Triton-X 100 in PBS), free-floating sections were incubated overnight at 4°C with the desired primary antibody in 0.1% BSA/0.2% Triton-X 100 at 4°C. On the subsequent day, sections were incubated for 75 min in the correspondent secondary antibody diluted in 0.1% BSA. Tissue was washed in PBS 3 times and then rinsed, mounted, dehydrated, and cover-slipped with fluorsave (Calbiochem, San Diego, CA, USA). For tridimensional reconstruction of microglial cells, immunofluorescence for ionized calcium-binding adapter molecule 1 (Iba-1 1:200 – Merck- Millipore, Darmstadt, Germany) and DAPI (1:5,000) conjugated with cy3 (1:600 – Neomarker-Invitrogen, Fremont, CA, USA) was performed. Regarding astrocytes, polyclonal rabbit anti-glial fibrillary acid protein (GFAP 1:500 - DAKO, Glostrup, Denmark) counterstained with DAPI (1:5,000) and coupled with alexa 594 (1:500 – Neomarker-Invitrogen, Fremont, CA, USA) was used.

### Morphometric Analysis of Glial Cells

For tridimensional reconstruction of glia, two coronal sections *per* animal were stained for Iba-1 and DAPI (cell nuclei) for microglial cells or GFAP and DAPI for astrocytes, as described before. Sections were visualized using a Leica SP8 Confocal (Leica Microsystems, Germany), applying LASX software (version 3.1.5.1). Previews of the whole section in widefield (10X/0.3 dry) using the DAPI channel were taken to choose areas of interest in the ventral CA1 *stratum radiatum*, that was further acquired at a higher resolution with the confocal mode. Two channel (Iba-1/GFAP and DAPI) Z-stack images (Z-step intervals of 0.3 μm) were acquired using a 63X/1.4 oil objective and a DFC365 FX CCD Camera (Leica) with a x-y sampling of 72 nm. Cells were eligible for reconstruction if the following criteria were met: (i) the Iba-1/GFAP positive cell coincided with a single DAPI-stained nucleus; (ii) the cell did not present truncated processes; (iii) the cell could be singled out from neighboring cells to ensure correct reconstruction. A total of 75 cells (5 ROIs *per* animal) were selected for reconstruction performed using simple neurite tracer plugin available in FIJI-ImageJ software (v2.0.0), an open-source tool previously described to effectively assess tridimensional morphology of neurons and glial cells (53). Glia morphometric properties were evaluated by quantifying the number of processes, total length (in μm), sum of intersections; Sholl analysis was also performed to identify the number of intersections at radial intervals of 2 μm starting from the central point of the soma, as a measure of the complexity of glial cells ramifications and branches.

### Statistical Analysis

Quantitative results were analyzed using Student *t* and Mann–Whitney tests and ANOVA. The normal distribution of samples was checked with Shapiro–Wilks test and the homogeneity of variances was evaluated with *F* test. When the equal variance criterion was violated, the Welch correction was used. The Mann–Whitney non-parametric test was chosen when data were not normally distributed. Otherwise, Student *t-*test was used. All statistical tests were performed in Origin 9.0 (OriginLab Corporation, Northampton, MA, USA), except the morphology experiments for which statistical analysis was performed using Prism 8.2 (GraphPad Software Inc., San Francisco, CA, USA). The format of Student *t*-test results is: *t*(df) =*t* statistic, *p* significance value. The format of Mann–Whitney test results is: *U* (n1, n2) = x, *p* ≤ significance value. The format for ANOVA test was *F*(df)= *F, p* significance value. The tests are two-sided and confidence interval was set at 95% (0.95) so that the difference between means was considered statistically significant at *p*-values of <5% (0.05), 1% (0.01), and 0.1% (0.001). Data are shown as mean ± standard deviation (SD).

## Results

**Figure 2:**
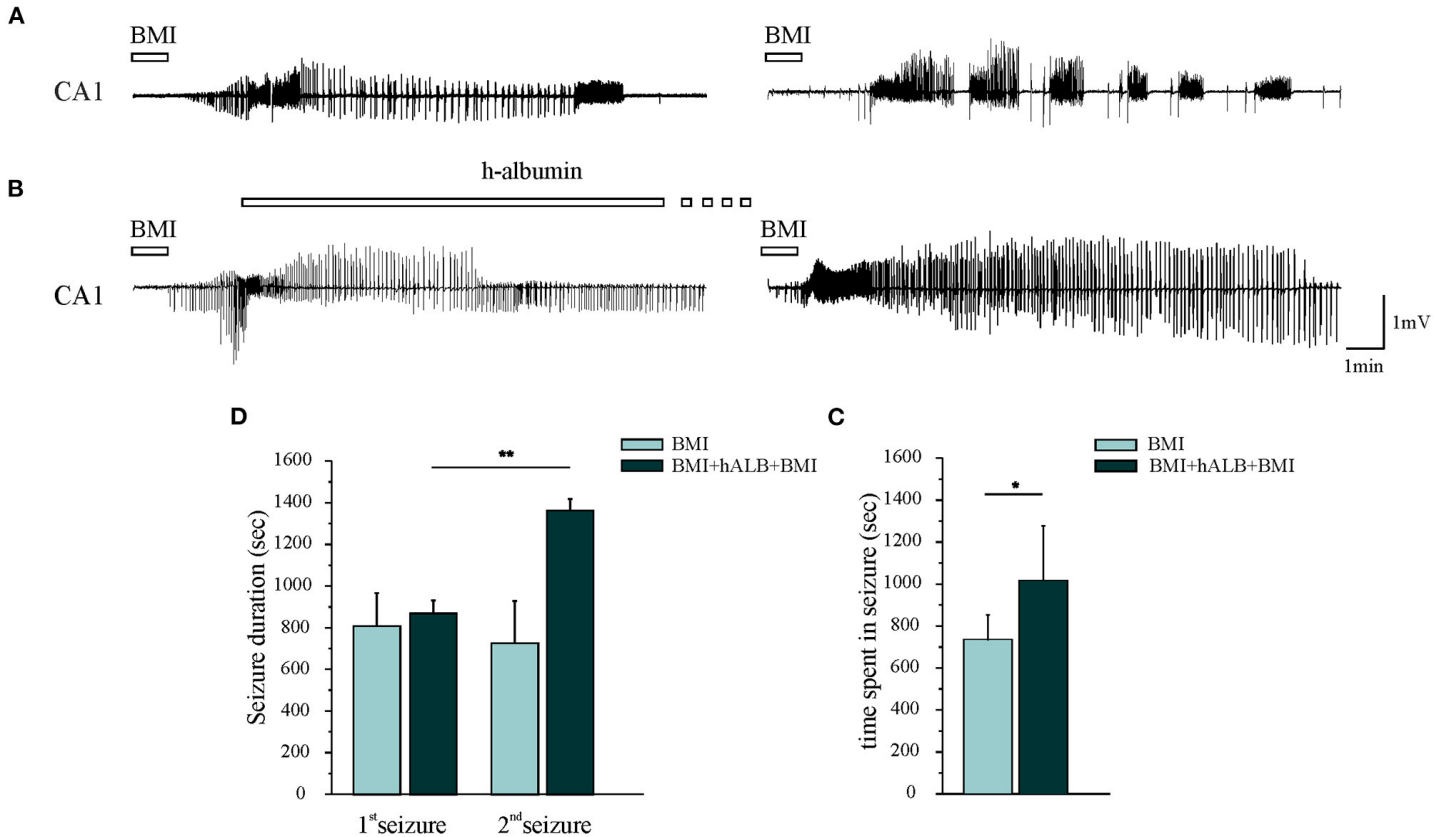
Epileptiform discharges recorded in CA1. **(A)** The first and the second 3-min application of BMI induced one seizure-like event (SLE). **(B)** 30-min h-recombinant albumin (h-ALB) perfusion after the first BMI-induced SLE (left trace) significantly increased the duration of the second BMI-induced SLE (right trace). **(C)** Mean SLE durations induced by the first (1st SLE) and the second application of BMI with and without h-recombinant albumin application (2nd SLE). **(D)** Total time spent in seizure after the application of the experimental protocols B and C, respectively. *p < 0.05; **p < 0.01 with Student *t*-test.

**Figure 3:**
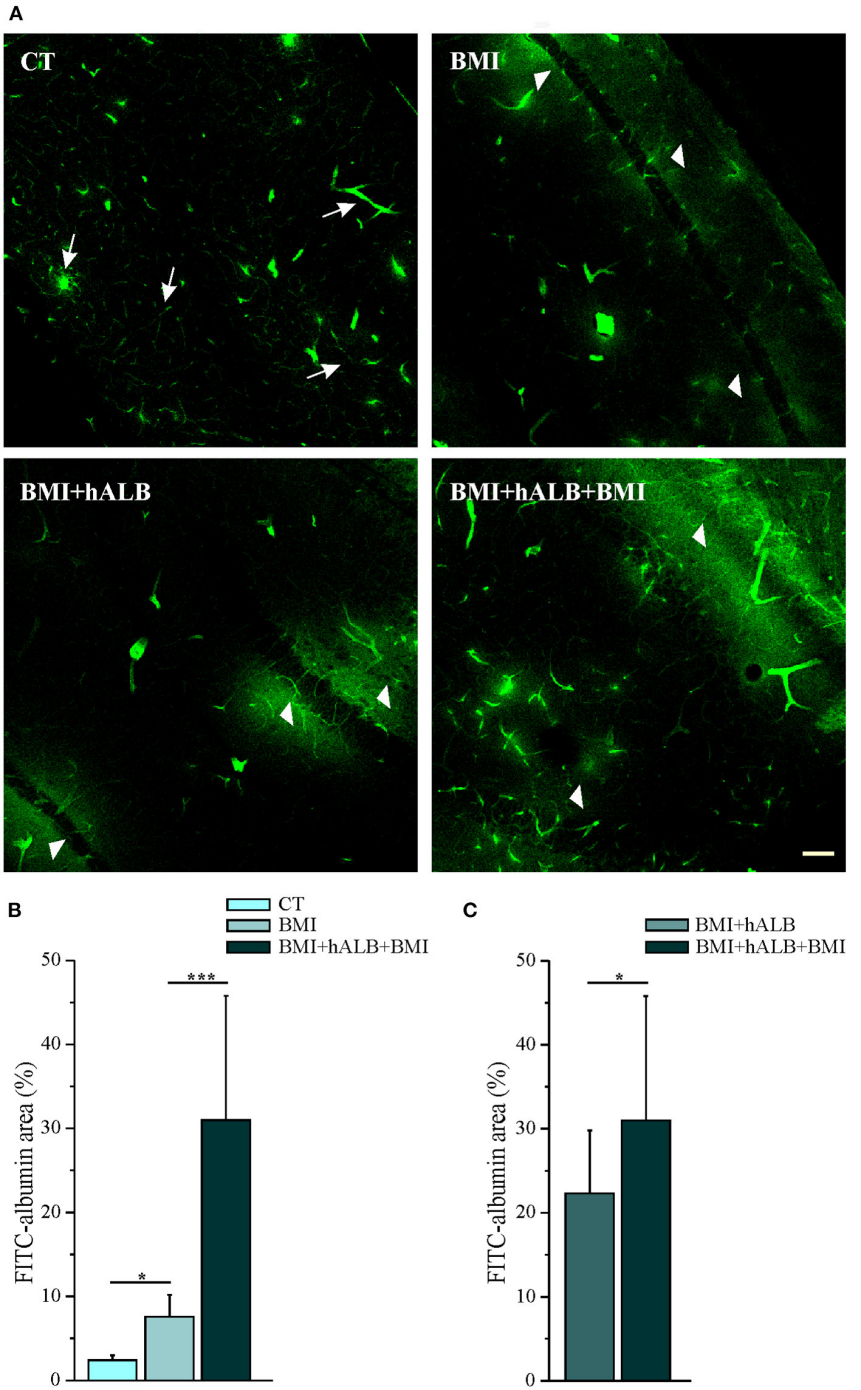
Quantification of parenchymal Fluorescein isothiocyanate (FITC)–albumin leakage. **(A)** Representative photomicrographs of intraparenchymal FITC–albumin signal in the limbic area in control solution (top, left), BMI (top, right), BMI + h-ALB (bottom, left), and BMI + h-ALB + BMI (bottom, right) treated brains. Control sections show intraluminal signal with scattered perivascular spots (white arrow). Areas of FITC–albumin parenchymal extravasation around vessels (white arrowheads) after the second pulse of BMI alone or co-perfused with h-recombinant albumin, showed as FITC–albumin parenchymal leakage is broader after the application of protocol C and D (see Figure 1). **(B,C)** Quantification of parenchymal FITC–albumin leakage in the experimental conditions. FITC-albumin leakage has been evaluated as spot area (number of pixels) and it is expressed as percentage of values vs. control experiments *p < 0.05; ***p < 0.001 with ANOVA and Student *t*-test. Calibration bar = 100 μm.

Experiments were performed in 15 isolated guinea pig brains. Control condition brains were maintained *in vitro* with control solution for 4 h before perfusing FITC-alb (Figure 1A). The reasoning behind the 4 h timeline was due to technical issues. The isolated *in vitro* brain takes 90 min (0.2°C/ min) to reach 32°C, which is the optimal temperature for the experiments to be carried on. Subsequently, LOT-evoked potentials were induced to verify the viability of the preparation throughout the experiment and assess the position of depth electrodes. The infusion of the first bolus of bicucculline followed. A second perfusion of bicuculline was applied 90 min after the first and the recording were carried on for 30 min. In the end, the brain was perfused for 4 min with FITC albumin. As expected, no SLEs were observed in control experiments (*n* = 4). SLEs were induced by arterial perfusion of BMI (50 μM) for 3 min. The first BMI application evoked a 13.5 ± 2.6 min SLEs in the limbic region, recorded in the hippocampus (area CA1; left trace in Figure 2A) and in the mEC. A second BMI perfusion applied 90 min after the first one (Figure 1B) induced SLEs lasting 12.1 ± 3.4 min (*n* = *4*; see Figure 2C). This protocol induced significant brain extravasation of FITC-albumin compared to control animals (Figures 3A,B; *t*(18) = −5, 5; *p* < 0.001 with two samples Student *t-*test). The increase in BBB permeability induced by a first SLE allowed extravasation of later perfused FITC-alb (49). Therefore, we perfused 4 g/L h-ALB via the basilar artery for 30 min immediately after the occurrence of the first BMI-induced SLE (*n* = 4; Figure 1C) and 60 min before the second BMI bolus (*n* = 4; Figure 1D). The perfusion of h-ALB increased both SLE duration induced by the second BMI perfusion (Figures 2B,C; 22.7 ± 0.9 min; t(5) = −4, 2; *p* < 0.01 with two sample student *t-*test) and the total time spent in SLE (Figure 2D; *t*(12)= −2, 4; *p* < 0.05 with two sample student *t-*test) compared to the experiments without h-ALB perfusion between the two BMI tests. As expected, the extent of BBB leakage, assessed by measuring the area of FITC-alb extravasation, was up to 3-fold larger after BMI + hALB + BMI perfusion compared to BMI only (Figures 3A,B; *F*(2) = 60; *p* < 0.001 with ANOVA). Also, the extent of BBB leakage induced by application of BMI +hALB was lower compared to BMI+hALB+BMI (Figures 3A,C; *t*(23) = 2, 3; *p* < 0.05 with two samples Student *t-*test).

**Figure 4:**
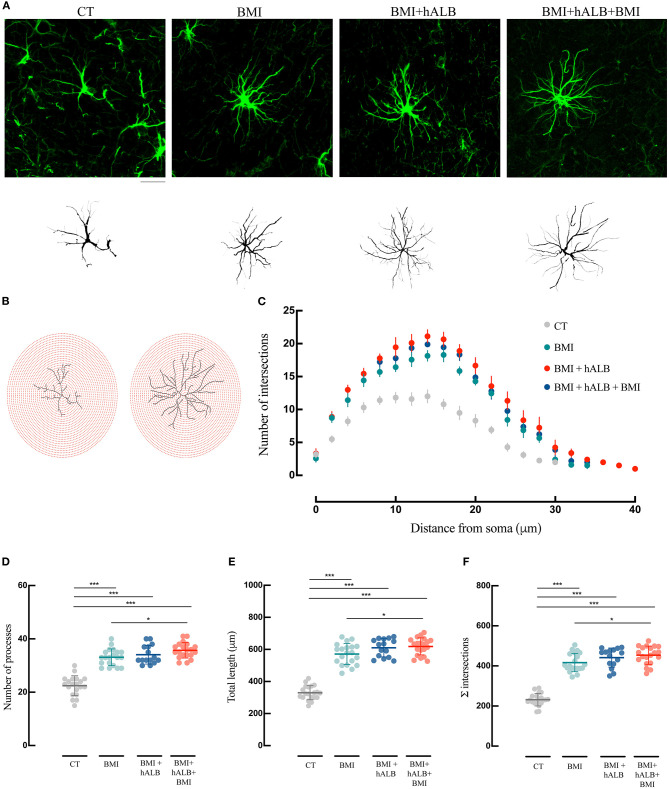
Morphological analysis and reconstruction of astrocytes in ventral hippocampal CA1. **(A)** Representative morphologies of astrocytes in GFAP immunofluorescence coronal sections of the ventral CA1 hippocampal region are shown for CT, BMI, BMI+hALB, and BMI+hALB+BMI; the correspondent reconstruction of the astrocyte is illustrated in the lower part of the panel. Calibration bar = 20 μm. **(B)** Representative Sholl analysis setting of a manually reconstructed astrocyte from CT (left) and BMI+hALB+BMI (right). The circles centered around the soma are separated by radial intervals of 2 μm. **(C)** Number of intersections *per* 2 μm radius plotted against the distance from the cell soma in CT, BMI, BMI+hALB, and BMI+hALB+BMI animals. **(D)** Number of processes *per* 2 μm radius of astrocytes in CT (*n* = 20 cells), BMI (*n* = 20 cells), BMI+hALB (*n* = 15 cells), and BMI+hALB+BMI (*n* = 20 cells) animals. **(E)** Total length in μm of astrocytes in CT (*n* = 20 cells), BMI (*n* = 20 cells), BMI+hALB (*n* = 15 cells), and BMI+hALB+BMI (*n* = 20 cells) guinea pigs. **(F)** Sum of intersections of astrocytic cells CT (*n* = 20 cells), BMI (*n* = 20 cells), BMI+hALB (*n* = 15 cells), and BMI+hALB+BMI (*n* = 20 cells) animal groups. *p < 0.05; ***p < 0.001 with ANOVA test. All experiments were done in 5 cells *per* animal.

**Figure 5:**
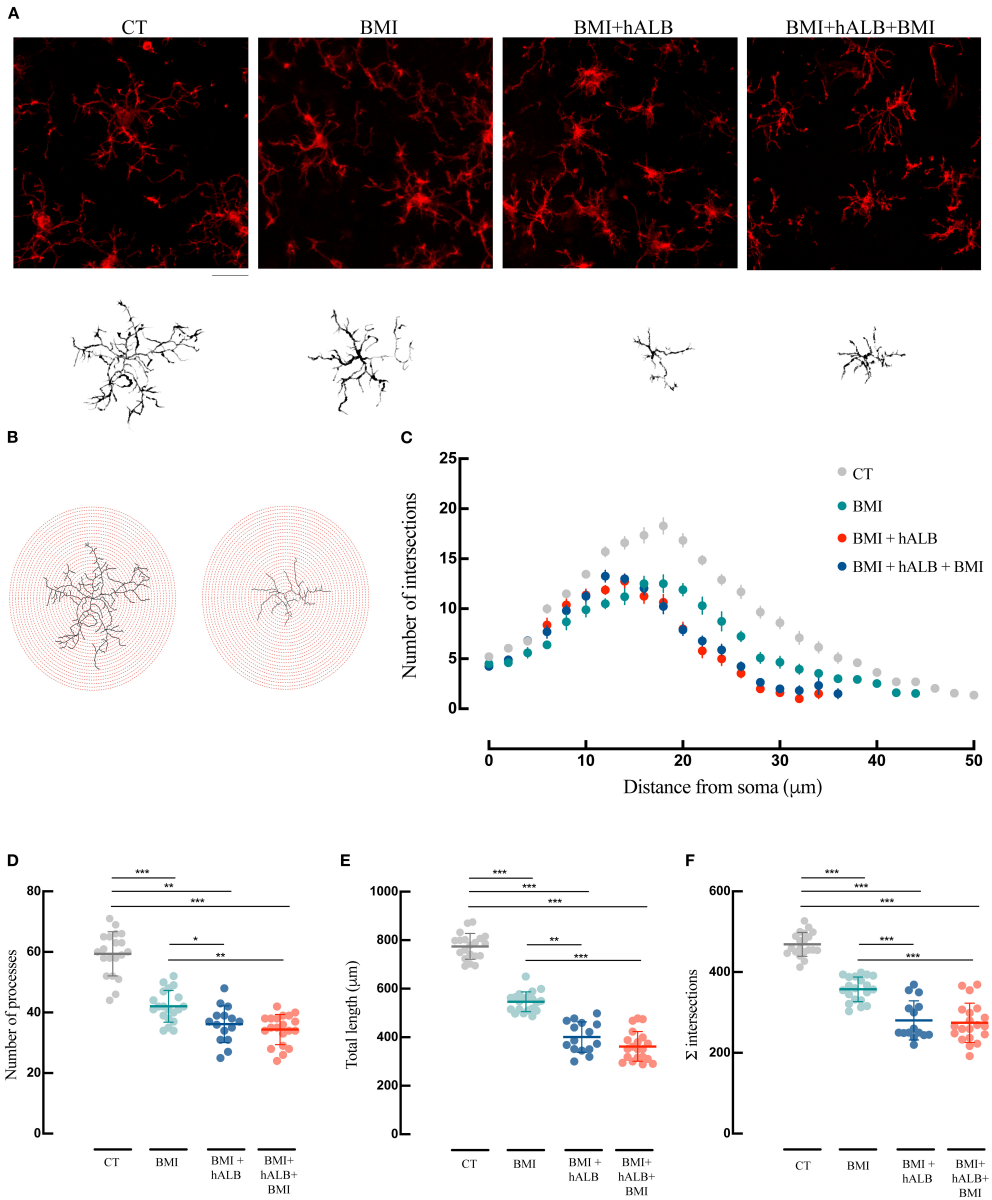
Morphological analysis and reconstruction of microglia in ventral hippocampal CA1. **(A)** Representative morphologies of microglial cells in Iba-1 immunofluorescence coronal sections of the ventral CA1 hippocampal region are represented for CT, BMI, BMI+hALB, and BMI+hALB+BMI; the correspondent reconstruction of the microglial cell is illustrated in the bottom part of the panel. Calibration bar = 20 μm. **(B)** Representative Sholl analysis setting of a manually reconstructed microglia cell from CT (left) and BMI+hALB+BMI (right). The circles centered around the soma are separated by radial intervals of 2 μm. **(C)** Number of intersections *per* 2 μm radius plotted against the distance from the cell soma in CT, BMI, BMI+hALB, and BMI+hALB+BMI animals. **(D)** Number of processes *per* 2 μm radius of microglia cells in CT (*n* = 20 cells), BMI (*n* = 20 cells), BMI+hALB (*n* = 15 cells), and BMI+hALB+BMI (*n* = 20 cells) guinea pigs. **(E)** Total length in μm of microglia cells in CT (*n* = 20 cells), BMI (*n* = 20 cells), BMI+hALB (*n* = 15 cells), and BMI+hALB+BMI (*n* = 20 cells) animal groups. **(F)** Sum of intersections of microglia cells in CT (*n* = 20 cells), BMI (*n* = 20 cells), BMI+hALB (*n* = 15 cells) and BMI+hALB+BMI (*n* = 20 cells) animals. *p < 0.05; **p < 0.01; ***p < 0.001 with ANOVA test. All experiments were done in 5 cells *per* animal.

To exclude unspecific effects, in 2 experiments h-ALB was perfused via the basilar artery for 30 min without BMI. H-ALB perfusion alone was unable to spontaneously evoke ictal discharge (data not shown). Afterwards, we evaluated the influence of SLE activity either alone (BMI) or in combination with h-ALB (BMI + hALB) on the reactive state of GFAP immunostained astrocytes (Figure 4) and IBA-1 stained microglial cells (Figure 5) analyzed in the CA1 hippocampal field, where epileptiform activity was recorded. In order to better investigate the role of serum albumin on glial dysfunction and BBB damage, in a separate set of experiments we also studied the effect of h-ALB after the first BMI-induced SLE. In this case, at the end of the h-ALB treatment, the isolated brain was perfused with perfusion solution until the end of the experiment (*n* = 3). Sholl analysis was used to quantify the number of intersections at radial intervals of 2 μm starting from the soma of glial cells (Figures 4B,C, 5B,C). As summarized in Figure 4C, the number of intersections counted in CA1 astrocytes from guinea pig brains submitted to BMI, BMI + hALB and BMI + hALB +BMI was higher than control brains (*F*(3) = 115.9; *p* < 0.001 with ANOVA). Representative astroglia typical of the four experimental conditions are illustrated in Figure 4A. Furthermore, astrocytes had a higher number of processes (Figure 4D), total length of their processes (Figure 4E), and sum of intersections in Sholl analysis (Figure 4F) in BMI, BMI + hALB, and BMI + hALB + BMI in comparison to CT animals (*F*(3) = 65.18, *F*(3) = 119.3, and *F*(3) = 115.9, respectively; *p* < 0.001 with ANOVA). Interestingly, there was a consistent increase in all three parameters when comparing BMI against BMI + hALB + BMI (Figures 4D–F), indicating that the astrocitic morphological changes that occured in the presence of SLEs only (BMI) worsened in the BMI + hALB + BMI protocol.

Lastly, microglial morphology was also assessed (Figure 5) using the same methodology employed for astrocyte reconstruction. Sholl analysis revealed that microglia in BMI, BMI + hALB, BMI + hALB + BMI animals had a lower number of intersections when compared to control animals (*F*(3) = 99.2; *p* < 0.001 with ANOVA; Figures 5B,C – representative panel in Figure 5A). Moreover, microglia consistently had lower number of processes (Figure 5D), total length of their processes (Figure 5E) and sum of Sholl-analysis intersections (Figure 5F) in BMI, BMI + hALB, and BMI + hALB + BMI animal cohorts, in comparison to control brains (*F*(3) = 71.18, *F*(3) = 225.9 and *F*(3) = 99.2, respectively; *p* < 0.001 with ANOVA). Similar to astrocytes, when BMI was coupled with h-ALB + BMI, the morphology of microglia had a more activated phenotype when compared to BMI alone (Figures 5D–F). Additionally, comparing microglia cells that had the same seizure profile (single seizure induced by BMI), cells that were perfused with hALB as well had a higher gliosis-like phenotype when compared to BMI only protocol (BMI vs. BMI + hALB in Figures 5D–F). In sum, our data demonstrate a worsening of the astro and microgliosis state when major seizure activity is in coallition with h-ALB extravasation into the brain. However, h-ALB by itself is also able to induce major gliosios (to a less extent than the previous mentioned protocol).

## Discussion

A growing body of evidence supports gliosis as a primary factor in the pathogenesis of neurological diseases (54, 55). Data from human and animal studies support the notion that glial cells contribute to the control of neuronal function under both physiological and pathological conditions (11, 12, 56) and respond to changes in normal physiology of the CNS by establishing and coordinating response to disease resulting in gliosis (57). Recent evidences from experimental models of epilepsy and drug-resistant forms of human epilepsy suggest that epilepsy is often accompanied by astrocytes and microglia phenotypic and functional alterations (12, 21, 58).

We previously demonstrated that pharmacologically-evoked SLEs in the *in vitro* guinea pig brain induce IL-1β expression in perivascular astrocytes and compromise BBB permeability (44, 49). Our data confirmed that serum albumin entering into the brain through an impaired BBB contributed to the generation of sustained epileptiform activity (49). In the present study we investigated the role of serum albumin extravasation into brain parenchyma following seizure-induced BBB damage in enhancing reactive gliosis without the contribution of any blood-borne molecules/cells, since our guinea pig brain preparation is maintained in isolation. The BBB is involved in almost all pathologies of the CNS (59–61). Its alterations can compromise the fundamental processes which govern brain functions. Serum albumin extravasation into brain parenchyma following BBB integrity loss is reported to lead to glial activation and alterations in the extracellular milieu around neurons (8, 62). Normal brain albumin concentration is much lower (35–50 microg/mL) than blood albumin concentration, that ranges from 35 to 50 mg/mL (63, 64). Thus, BBB opening has the potential to expose brain cells to high levels of albumin (65). The contribution of serum albumin in astrocytes activation is supported by several studies showing induction of calcium signaling and DNA synthesis in astrocytes (66, 67). One pivotal mechanism involved in these effects is the albumin-mediated activation of the *transforming growth factor beta receptor II* (TGF-βR); recent studies demonstrated that serum albumin leaks into brain parenchyma through a dysfunctional BBB to bind astrocytic TGF-βR activating TGF-β signaling (8, 9, 68, 69). This cascade of events leads to astrocytes Kir4.1 downregulation and to their consequent failure to buffer extracellular K^+^ and glutamate, that culminates in the synthesis of inflammatory molecules and increase brain excitability (9, 56). Accordingly, blockade of Kir4.1 in glia with cesium has been demonstrated to promote seizure like activity (70). Furthermore, activation of TGF-β signaling by albumin induced rapid and persistent up-regulation of genes related to inflammation (9). BBB impairment also easily allows microglia to be exposed to high concentrations of albumin. Even though the effects of albumin on cells in the brain have mainly been investigated in astrocytes, several studies support the pathological role of microglial activation by albumin (63, 69, 71). Since albumin can activate microglia, which in turn can activate astrocytes and exacerbate reactive pathways (40, 41), it is of the upmost interest to understand which signaling cascades are activated in microglia exposed to serum albumin after BBB damage. Hooper and colleagues demonstrated that microglia respond to serum albumin by increasing intracellular calcium *via* Src tyrosine kinases, which successively leads to glutamate and TNF-α release (63, 65).

In our experiments, the concentration of albumin perfused in the arterial system of the *in vitro* guinea pig brain (4 mg/ml) falls within the range associated with BBB damage occurring in a pathology associated condition (65). The changes observed in our acute ictogenic model confirmed that astro- and microglial cells promptly respond to seizure activity. BMI-evoked SLE determined changes in astrocytic and microglial morphological phenotype toward a more activated state. SLEs-induced microglia adopts an amoeboid shape, starting from a ramified structure in the control brains (72, 73), while astrocytes express a hypertrophic phenotype with longer processes compared to control condition (74). Interestingly, seizure pattern, duration and astro- and microgliosis were exacerbated when SLE activity was combined with the perfusion of h-albumin. Our data support the hypothesis that albumin increases SLE activity in limbic areas by directly inducing a reactive state in both astrocytes and microglia. Whether acute gliosis represents an early possible defensive mechanism triggered by seizure activity or their activation is actively involved in the epileptogenic process cannot be answered in our acute experimental conditions. However, *in vivo* studies performed in our laboratory in the intrahippocampal kainic acid (KA) model suggest that seizures, gliosis and BBB damage contribute to epileptogenesis at the site of kainic acid injection, but not in regions remote from the injection site. Even though gliosis was still present at an early phase and seizure activity was present in both regions, no detrimental markers of brain damage were detected (75). In the same animal model, genes associated with inflammatory response (IL1-β and COX-2), brain activity (c-FOS) and oxidative stress (HO-1) were early upregulated exclusively in the KA-injected hippocampus during the acute phase and remained upregulated 1 month post-KA injection. Interestingly, only genes linked to glial function (AQP4 and Kir4.1) were upregulated 3 days post-KA (but not after 1 month) in regions remote from the kainic acid injection site that also generated epileptiform discharges. In these regions late damage did not develop (Vila Verde in press on Neurophatolo Appl Neurobiol). It can be hypothesized that early after seizure occurrence transient gliosis could helping neurons to cope seizure activity preventing neuronal damage, whereas in regions in which seizures are coupled with the excitotoxic effects of kainic acid, persistent gliosis may have nefarious effects to the brain. It can therefore be expeculated that, early after seizure occurrence, transient gliosis may help neurons cope with seizure activity preventing neuronal damage development, whereas in regions in which seizures are coupled with the excitotoxic effects of kainic acid, persistent gliosis induces permanent nefarious effects in the brain.

In conclusion, the present study reinforces our previous observation that in an *in vitro* acute model of ictogenesis seizure activity *per se* enhances BBB permeability in brain regions involved in seizure generation and that extravasation of albumin into brain parenchyma increases seizure activity in those regions affected by BBB impairment (44, 49). We demonstrate for the first time simultaneous morphological phenotype changes in both astrocytes and microglia due to seizure activity. Our data strongly suggest seizure-induced BBB breakdown and the consequent albumin extravasation leads to astrocytes and microglia reactivity and eventually to reinforce seizure activity by increasing its duration. Further studies are required to recognize when astro- and microgliosis might help or harm the brain in our experimental conditions.

## Data Availability Statement

The original contributions presented in the study are included in the article/supplementary materials, further inquiries can be directed to the corresponding author.

## Ethics Statement

The animal study was reviewed and approved by Organismo Preposto al Benessere Animale - OPBA Fondazione Istituto Neurologico C. Besta Via Celoria 11 20133 Milano.

## Author Contributions

DV and LL: conception, design of the study, acquisition, and analysis of data. DV, LL, and MC: drafting a significant portion of the manuscript or figures. All authors contributed to the article and approved the submitted version.

## Conflict of Interest

The authors declare that the research was conducted in the absence of any commercial or financial relationships that could be construed as a potential conflict of interest. The handling editor is currently organizing a Research Topic with one of the authors MC.
